# Evaluation of Waste Mushroom Compost as a Feed Supplement and Its Effects on the Fat Metabolism and Antioxidant Capacity of Broilers

**DOI:** 10.3390/ani10030445

**Published:** 2020-03-06

**Authors:** Wen Yang Chuang, Chu Ling Liu, Chia Fen Tsai, Wei Chih Lin, Shen Chang Chang, Hsin Der Shih, Yi Ming Shy, Tzu-Tai Lee

**Affiliations:** 1 Department of Animal Science, National Chung Hsing University, Taichung 402, Taiwan; xssaazxssaaz@yahoo.com.tw (W.Y.C.); s04610215@gmail.com (C.L.L.); bbella1123tw@gmail.com (C.F.T.); waynezi2227738@gmail.com (W.C.L.); 2 Kaohsiung Animal Propagation Station, Livestock Research Institute, Council of Agriculture, Tainan 71246, Taiwan; macawh@mail.tlri.gov.tw; 3 Taiwan Agricultural Research Institute Council of Agriculture, Executive Yuan, Taichung City 41362, Taiwan; tedshih@tari.gov.tw; 4 Hsinchu Branch, Taiwan Livestock Research Institute, Council of Agriculture, Executive Yuan, Tainan 71246, Taiwan; emshy@tlri.gov.tw; 5 The iEGG and Animal Biotechnology Center, National Chung Hsing University, Taichung 402, Taiwan

**Keywords:** mushroom compost, fat metabolism, antioxidant, broilers

## Abstract

**Simple Summary:**

Mushroom waste compost is the main byproduct when cultivating mushrooms. Due to its high mycelium content, mushroom waste compost may improve animal health by increasing antioxidant capacity. Furthermore, increasing evidence suggests that supplementing animal diets with fiber could improve body composition and health. The results showed that supplementation with mushroom waste compost accelerates adipolysis and enhances the antioxidant capacity of broilers. Among all treatment groups, broilers given dietary supplementation with 0.5% mushroom waste compost showed improved feed conversion rate and the highest adipose metabolism.

**Abstract:**

*Pennisetum purpureum* Schum No. *2* waste mushroom compost (PWMC) is the main byproduct when cultivating *Pleurotus eryngii*. Due to the high mycelium levels in PWMC, it may have potential as a feed supplement for broilers. This study investigated the effects of PWMC supplementation on antioxidant capacity and adipose metabolism in broilers. In the study, 240 broilers were randomly allocated to one of four treatment groups: basal diet (control), 0.5%, 1%, or 2% PWMC supplementation. Each treatment group had 60 broilers, divided into three replicates. The results showed that supplementation with 0.5% PWMC decreased the feed conversion rate (FCR) from 1.36 to 1.28, compared to the control. Supplementation with 0.5% or 2% PWMC decreased glucose and triglyceride levels, compared to the control (*p* < 0.0001), the concentrations of adiponectin and oxytocin increased from 5948 to 5709, 11820, and 7938 ng/ mL; and 259 to 447, 873, and 963 pg/ mL, respectively. Toll-like receptor 4 was slightly increased in the 0.5% and 1% PWMC groups. Both interferon-γ (IFN-γ) and interleukin-1ß (IL-1ß) were significantly decreased, by about three to five times for IFN-γ (*p* < 0.0001) and 1.1 to 1.6 times for IL-1ß (*p* = 0.0002). All antioxidant-related mRNA, including nuclear factor erythroid 2–related factor 2 (Nrf-2) and superoxidase dismutase-1 (SOD-1), increased significantly following PWMC supplementation. Both claudin-1 and zonula occludens 1 increased, especially in the 2% PWMC group. Excitatory amino acid transporter 3 (EAAT3) significantly increased by about 5, 12, and 11 times in the 0.5%, 1%, and 2% PWMC groups. All adipolysis-related mRNA were induced in the PWMC treatment groups, further enhancing adipolysis. Overall, 0.5% PWMC supplementation was recommended due to its improving FCR, similar antioxidant capacity, and upregulated adipolysis.

## 1. Introduction

Due to global climate change and rising crude oil prices, high-starch animal feed ingredients such as corn and cassava must be reconsidered in view of their value as raw materials for biomass energy [1]. Considering the cost of raising broilers, it is increasingly important to find cheap alternatives that can maintain animal growth and health. Crops are the main sources of raw feed materials, resulting in a large amount of high-fiber agricultural byproducts [2]. Traditionally, due to the limitations of animal digestion, high-fiber plant materials have not been considered for animal feed [3]. Unlike animals, mushrooms produce a large amount of cellulase and hemicellulase, and fiber is the main source of their growth [4]. As such, the culture medium used in mushroom cultivation is high in fiber and contains few minerals or crude proteins [4]. However, following cultivation, the medium is still high in crude fiber and may cause new environmental issues [5,6].

Due to its high-fiber characteristics, *Pennisetum* can be used as one of the raw materials or an adjustment material in the cultivation of mushrooms, reducing the environmental problems caused by the traditional use of wood chips [6]. In addition, mushroom waste compost produced by mushroom planting contains a lot of mycelium, which could improve animal antioxidant capacity and health [5]. Mahfuz et al. also indicated that although 2% mushroom waste compost addition would not increase the feed conversion rate or body weight of broilers, mushroom waste compost addition could decrease broilers’ serum cholesterol content and improve immunomodulation [7].

In addition, previous researches pointed out that plant-based phenol-like compounds, such as epigallocatechin gallate (EGCG), could suppress fatty acid synthase and acetyl CoA carboxylase activities thereby decreasing the adipogenesis in broilers [8]. Lee et al. [9] also pointed out that the addition of mushroom waste compost could decrease crude fat in broiler meat and the results might be caused by the decrease of lipid peroxidation.

However, there is limited research on the reuse of mushroom waste compost or its effects when used as a supplement in broiler feed. This study therefore investigated the effect of mushroom waste compost on antioxidant capacity and how could it alter adipose metabolism in broilers.

## 2. Materials and Methods

### 2.1. The Collection and Characteristics of Pennisetum purpureum Schum No. 2 Waste Mushroom Compost (PWMC)

PWMC was the medium used to grow *Pleurotus eryngii*. It contained at least 70% *Pennisetum purpureum* Schum No. 2 (PP) and all mature *P. eryngii* were removed. The PWMC came from the Taiwan Agricultural Research Institute Council of Agriculture, Executive Yuan. Briefly, after removing *P. eryngii*, the medium with fresh residues had about 50% moisture and was immediately dried at 50 °C for 1 day before being crushed into powder. PWMC was stored at 4 °C until use.

The Folin–Ciocalteu method described by Tabart et al. [10] was used for the total phenol content analysis. Briefly, the sample was extracted by deionized water at 95 °C for 30 min and cooled down to room temperature before being used. Sample solution of 1 mL was mixed with 5 mL Folin–Ciocalteu reagent and 4 mL 7.5% sodium carbonate. The mixture was incubated in the room temperature for 30 min and measured the absorbance at 730 nm. The 0.01–0.1 mg/mL gallic acid was also measured by the methods described above for the standard curve. The presentation of total phenol content was described as gallic acid equivalent (GAE) mg/g sample.

PWMC was extracted by deionized water at 95 °C for 30 min and cooled down to room temperature, and filtrated by a 0.22 μm filter before used for the phenol-like compounds detection. HPLC (Hitachi, Kyoto, Japan) equipped with a pump (CM 5110), a column (C18-AR, 250 × 4.8 mm, maintained at 40 °C by the column oven (CM 5310)), an autosampler (L-2200) and a computer system with HPLC D-2000 Elite. The mobile phase conditions were (A) 0.05% *v*/*v* H_3_PO_4_ and (B) 3:2 *v*/*v* CH_3_OH/CH_3_CN solution, 1.0 mL/min, and UV detection (CM 5420) at 280nm. Also, 0.01~1.5 mg/ mL of gallocatechin (GC), epigallocatechin (EGC), catechin (CC), epicatechin (EC), gallic acid (GA), epigallocatechin gallate (EGCG), epicatechin gallate (ECG), catechin gallate (CG), and caffeic acid were measured by the methods described above for the standard curve.

The methods of total flavonoid content were described by Pourmorad et al. [11]. Briefly, a sample was extracted by methanol on ice for 30 min. The 1 mL sample solution, 3 mL methanol, 0.2 mL 10% AlCl_3_, 0.2 mL 1M potassium acetate, and 5 mL distilled water were mixed and incubated at room temperature for 30 min. The absorbance of mixture was measured at 700 nm. Quercetin of 0.01–0.1 mg/mL was also measured by the methods described above for the standard curve.

The methods of crude triterpenoid content were described by Wei et al. [12]. Briefly, a 3 g sample was added to 100 mL of 50% ethanol and shaken (100 rpm) for 8 h at room temperature. The solution was centrifuged at 3000 rpm and the supernatant was removed to a new tube. Next, 100 mL 95% ethanol was added in the precipitate and shaken (100 rpm) for 12 h at room temperature. The new solution was centrifuged at 3000 rpm and the two supernatants were mixed. The new supernatant was filtrated by Advantec No.1 filter papers and stoved at 50 °C for three days. The 100 mL saturated NaHCO_3_ (pH 3.5) and ethyl acetate were used to wash the remains to remove the impurity. The ethyl acetate solution was collected and dried to constant weight. The dry remains weight was measured and calculated as the formula described as follows.

Crude triterpenoid content (%) = Dry remains weight/ Sample weight × 100%(1)

### 2.2. Animal Experiment Design

PWMC was used to test its effect on broiler fat metabolism, nutrient absorption, antioxidant function, and inflammatory regulation. The experiment was carried out at National Chung Hsing University, Taiwan and all protocol followed that of the Animal Care and Use Committee in NCHU (IACUC: 108-049). Every broiler could drink and eat freely and had enough active space (about 20 broilers/9 m^2^). If the chickens had become infected during rearing, they would have been quarantined and given antibiotics or medication at the discretion of the veterinarian, however every broiler remained healthy throughout the whole rearing period. Euthanasia was used to reduce broiler suffering. The methods for animal design, testing intestinal morphology, blood and serum characteristics, and mRNA expression were slightly modified from those of Chuang et al. [13]. Briefly, 240 male 1-day-old Ross 308 broilers were used for 35-day experiments and each was placed in one of four groups: basal diet (control), 0.5%, 1%, or 2% PWMC supplementation. Every group had 60 broilers with three replicates (20 per pen). The initial body weight of each chick was similar (48.0 ± 0.7 g/bird) among all groups. The temperature was kept at 33 °C for 1-day-old chicks then slowly decreased to 22 °C after 30 days. To achieve or exceed the nutritional requirements of the broilers, according to NRC 1994, all diets were recalculated and the proximate composition was analyzed according to the AOAC (2012) [14] to determine the gross energy and crude protein levels (Table 1). The starter diet was provided for the first 21 days, and the finisher diet provided for days 22–35. Body weight gain, feed intake, and feed conversion rate (FCR) were measured on 21- and 35-day-old broilers. On day 35, the ileum, abdominal fat, spleen, and liver were collected for qPCR analysis.

### 2.3. Serum Characteristics

At the end of the experiment, 5 mL whole blood was collected from the broilers (three from each pen, nine per treatment) and left to stand for 4 to 5 h at 4 °C before measurement. The samples were centrifuged at 3000 rpm for 10 min to separate the blood cells and serum, before the serum was frozen at −20 °C until use. The concentrations of oxytocin (OXT), corticosterone), tumor necrosis factor alpha (TNF-α), glutathione peroxidase (Gpx), superoxidase dismutase (SOD) A), and malondialdehyde (MDA) in the broiler serum were measured. All analysis methods followed the manufacturer’s protocol. Briefly, the protein analytic was bound by the antibody and the enzyme-linked immunosorbent assay reader was used to measure absorbance at wavelengths provided by the manufacturer. Other blood cells and serum characteristics were measured using the automatic biochemical analyzer (Hitachi, 7150 auto-analyzer, Tokyo, Japan).

### 2.4. Total RNA Isolation and qPCR

Total RNA isolation and qPCR were used to analyze mRNA expression in the liver, spleen, ileum, and abdominal fat. All mRNA was collected from 35-day-old broilers in each treatment group. The methods of total mRNA isolation followed the manufacturer’s protocol (SuperScript™ FirstStrand Synthesis System reagent, Thermo Fisher, Waltham, MA, USA). The mRNA purity was determined by the absorbance ratio of 260/280 nm, and the methods for cDNA synthesis and qPCR analysis were as per Chuang et al. [13] Briefly, cDNA was mixed with 2× SYBR GREEN PCR Master Mix-ROX (Gunster Biotech, Co., Ltd., Taipei, Taiwan), deionized water, and each primer at a ratio of 5:1.2:1.8:1. A StepOnePlus™ Real-Time PCR System (Thermo Fisher, Waltham, MA, USA) was used to detect qRT-PCR performance. The 2^−ΔΔCt^ method was used to calculate the relative mRNA expression level, and ß-actin was used as the housekeeping gene for normalization. All primer sequences matched the genes of *Gallus gallus* (chicken) from Genbank (Table 2).

### 2.5. Statistical Analysis

All data analysis was calculated using SAS software (SAS^®^ 9.4, 2018, SAS Institute Inc., Cary, NC, USA). The difference between each experimental group was determined by the Tukey range test with a P value less than 0.05. The chemical analysis value present in the unit of “%DM” means analysis value/the percentage of dry matter (DM).

## 3. Results

### 3.1. The Characteristics of PWMC

Table 3 reports the levels of the different functional compounds in PWMC, including crude triterpenes, polyphenols, and flavonoids. The total phenol and flavonoids content were 1.84 GAE mg/g DM and 1.20 QE mg/g DM in PWMC. Among the phenol-like compounds, the concentrations of GA, GC, EGC, CC, caffeic acid, EC, EGCG, ECG, and CG in PWMC were 114, 1035, 1493, 7.9, 113, 230, 210, 78, and 401 μg/g DM.

### 3.2. Broiler Growth Performance with PWMC Supplementation

Broiler growth performance for days 1–21 and 22–35 are shown in Table 4. Within these two evaluation periods, there was no significant difference in body weight, weight gain, and feed consumption. The best feed conversion rate (FCR) was seen in the 0.5% PWMC group during the starter stage, compared to the control (1.36 vs. 1.28, *p* = 0.0374). However, there were no significant changes in weight gain and FCR for days 22–35 and 1–35.

### 3.3. Intestinal Barrier and Nutrient Absorption-Related mRNA Expression in 35-Day-Old Broilers

**Figure 1:**
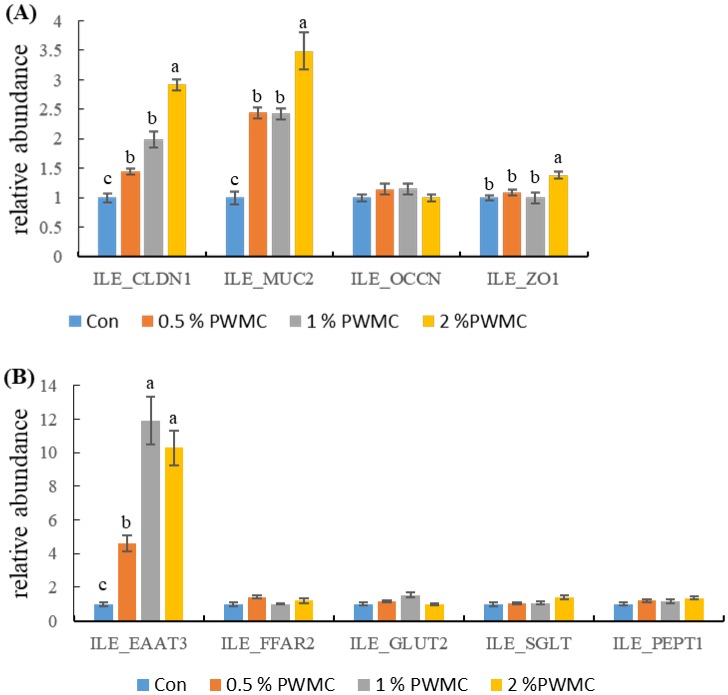
Intestinal barrier (**A**) and nutrient absorption-related (**B**) mRNA expression in the ileum (ILE) for 35-day-old broilers. CLDN: Claudin-1; MUC-2: Mucin2; OCCN: Occludin; ZO-1: Zonula occludens 1; EAAT3: Excitatory amino acid transporter 3; FFAR2: Free fatty acid receptor 2; GLUT2: Glucose transporter 2; SGLT: Sodium-dependent glucose cotransporter 1; PEPT-1: Peptide transporter 1. ^a-c^ Means within the same rows without the same superscript letter are significantly different (*p* < 0.05).

There was no significant difference in occludin (*OCCN*) content within each group (*p* > 0.05). However, mucin 2 (*MUC2*), the major protein that maintains the function and structure of mucus, increased about 2.5, 2.5, and 3.5 times in the 0.5%, 1%, and 2% PWMC groups, respectively (Figure 1A, *p* < 0.0001). Both claudin-1 (*CLDN-1*) and zonula occludens 1 (*ZO-1*), which are tight junction-related proteins, increased in the PWMC-supplemented groups, especially 2% PWMC (Figure 1A, *p* < 0.0001 and =0.0245, respectively). Among all the groups, only 2% PWMC supplementation enhanced *ZO-1* mRNA expression. The amount of PWMC supplementation was positively correlated to *CLDN1* mRNA expression but there was no significant difference between the 0.5% and 1% PWMC groups.

Most nutrient absorption is through transporters on the intestinal epithelial cells. By adding PWMC to broiler feed, excitatory amino acid transporter 3 (*EAAT3*) levels significantly increased by about 5, 12, and 11 times for the 0.5%, 1%, and 2% PWMC groups, respectively (Figure 1B, *p* < 0.0001). However, the expression of other nutrient absorption-related mRNA, like free fatty acid receptor 2 (*FFAR2*), glucose transporter 2 (*GLUT2*), sodium-dependent glucose cotransporter 1 (*SGLT*), and peptide transporter 1 (*PEPT1*), was not significantly different from the control (Figure 1B).

### 3.4. Intestinal Morphology in 35-Day-Old Broilers

The addition of PWMC did not significantly change the villus height or crypt depth in the jejunum or ileum of 35-day-old broilers, however the *Tunica muscularis* in the jejunum increased significantly by 1.47, 1.34, and 1.06 times in the 0.5%, 1%, and 2% PWMC groups (*p* = 0.0104) (Table 5). Only the 0.5% PWMC group was significantly different from the control in jejunum *Tunica muscularis* (*p* = 0.010), while the 2% PWMC group had a villus:crypt ratio about 1.22 times higher than the control.

### 3.5. Adipogenesis- and Adipolysis-Related mRNA Expression in the Liver and Adipocytes of 35-Day-Old Broilers

**Figure 2:**
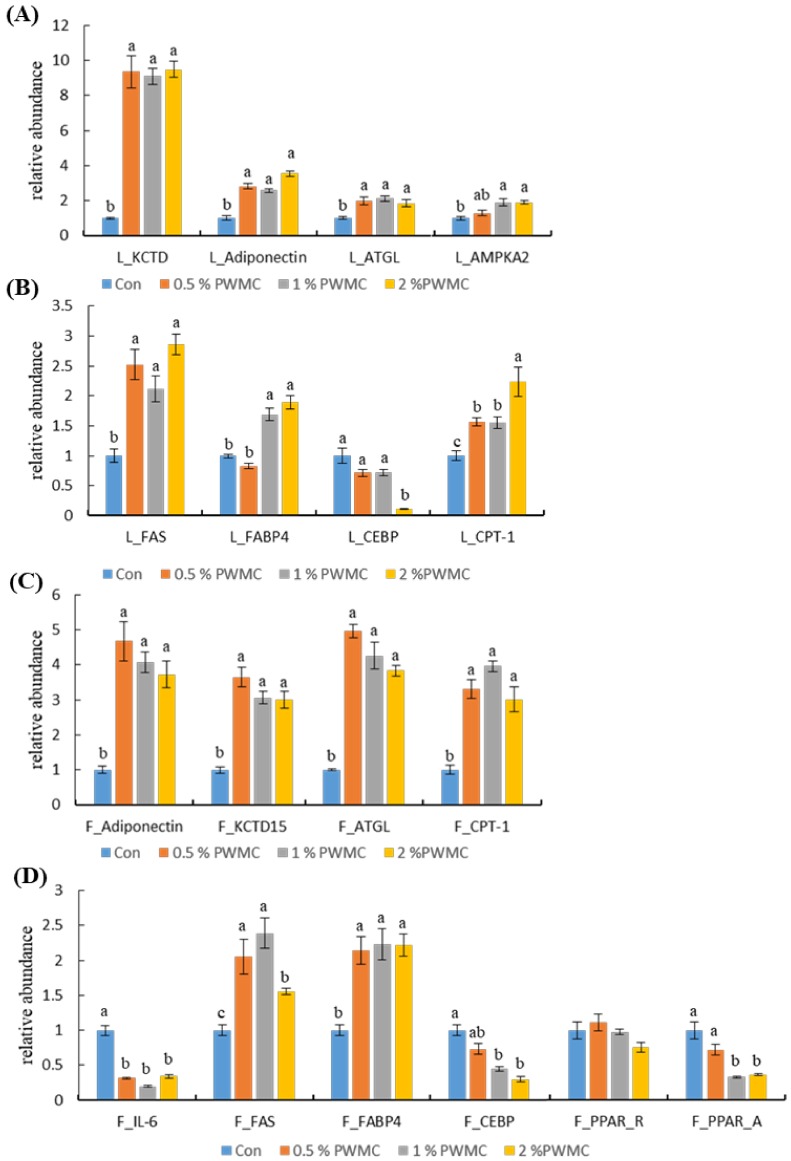
Adipolysis- and adipogenesis-related mRNA expression in the liver (L) and adipocytes (F) of 35-day-old broilers. (**A**) Potassium channel tetramerization domain-containing 15 (KCTD-15), adiponectin, adipose triglyceride lipase (ATGL) and 5′-AMP-activated protein kinase catalytic subunit alpha-2 (AMPK-α2) mRNA expression in L; (**B**) fatty acid synthase (FAS), fatty acid binding protein 4 (FABP4), CCAAT-enhancer-binding proteins-alpha (CEBPα), and carnitine palmitoyltransferase 1 (CPT-1) mRNA expression in L; (**C**) adiponectin, KCTD-15, ATGL, and CPT-1 mRNA expression in F; (**D**) interleukin-6 (IL-6), FAS, FABP4, CEBPα, peroxisome proliferator-activated receptor gamma (PPAR-γ), and peroxisome proliferator-activated receptor alpha (PPAR-α). ^a,b^ Means within the same rows without the same superscript letter are significantly different (*p* < 0.05).

The mRNA expression of potassium channel tetramerization domain-containing 15 (*KCTD-15*), adiponectin, adipose triglyceride lipase (*ATGL*), and 5′-AMP-activated protein kinase catalytic subunit alpha-2 (AMPK-α2) were significantly increased in the livers (L) of the treatment groups (*p* < 0.0001, < 0.0001, =0.0024 and = 0.0015, respectively), thereby increasing the rate of adipolysis (Figure 2A). There was no significant difference among the PWMC-supplemented groups. Similar results are shown in Figure 2C, where the adipolysis-related mRNA, including adiponectin, KCTD15, ATGL, and carnitine palmitoyltransferase 1 (*CPT-1*) (all of the *p* < 0.0001), increased in adipocytes (F) by at least four times. However, the adipogenesis-related mRNA shown in Figure 2B, including fatty acid synthase (*FAS*), fatty acid binding protein 4 (*FABP4*), and CCAAT-enhancer-binding proteins-alpha (*CEBPα*), had a compensatory one to three times increase in the adipocytes (*p* < 0.0001, <0.0001 and = 0.0052) (Figure 2D). When comparing adipogenesis- and adipolysis-related mRNA, the relative abundance of adipolysis-related mRNA expression was much higher (Figure 2). Nevertheless, interleukin-6 (IL-6) mRNA expression in adipocytes decreased (*p* < 0.0001), thereby decreasing inflammation.

### 3.6. Serum Characteristics in 35-Day-Old Broilers

As the data shows in Table 6, serum glucose content decreased in the 0.5% and 2% PWMC-supplemented groups (*p* < 0.0001), but there was no significant difference in MDA, SGOT, SGPT, or Alk-P levels. Gpx activity was not significantly different among the different groups (*p* = 0.6412), however SOD, the antioxidant-related enzyme, increased significantly from 523.9 mU/mL to 865.2 mU/mL, 842.6 mU/mL, and 879.3 mU/mL (*p* < 0.0001), respectively, and showed no dose effect. Serum TNF-α levels decreased 1.2 to two times (*p* = 0.001), and adiponectin increased the most in the 1% PWMC group (from 5948 ng/mL to 11820 ng/mL, compared to the control, *p* < 0.0001). OXT content also increased with a dose effect (*p* = 0.0016). The data showed that cholestenone (CHOL) levels, including high density lipoprotein-cholestenone (HDL-C) and low density lipoprotein- cholestenone (LDL-C), were not affected by the addition of PWMC, but triglyceride content decreased significantly (*p* < 0.0001).

### 3.7. Antioxidation and Inflammation-Related mRNA Expression in 35-Day-Old Broilers

**Figure 3:**
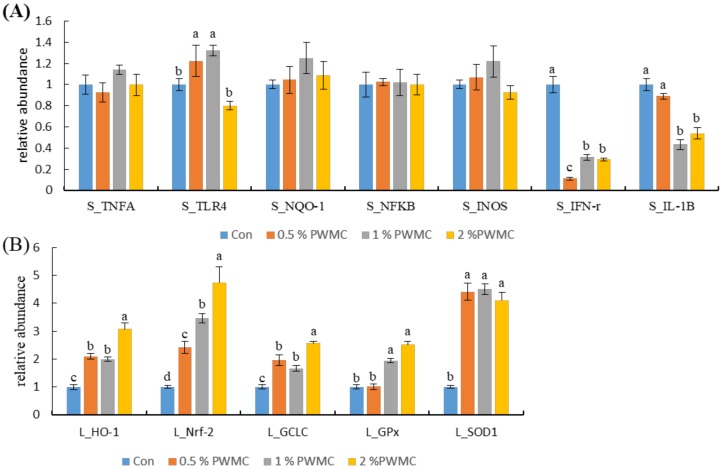
Inflammation- (**A**) and antioxidant-related (**B**) mRNA expression in the spleen (S) and liver (L) of 35-day-old broilers. TNF-α: Tumor necrosis factor alpha; TLR4: Toll-like receptor 4; NQO-1: NADPH dehydrogenase 1; NFκB: Nuclear factor kappa B p 65; iNOS: Inducible nitric oxide synthases; IFN-γ: Interferon-γ; IL-1ß: Interleukin-1ß; HO-1: Heme oxygenase-1; Nrf-2: Nuclear factor erythroid 2–related factor 2; GCLC: Glutamate-cysteine ligase catalytic; Gpx: glutathione peroxidase; SOD-1: Superoxide dismutase-1. ^a-d^ Means within the same rows without the same superscript letter are significantly different (*p* < 0.05).

When testing for inflammation-related mRNA expression in the spleens of 35-day-old broilers, there were no significant differences in TNF-α, NADPH dehydrogenase 1 (*NQO-1*), nuclear factor kappa B p 65 (*NFκB*), and inducible nitric oxide synthase (*iNOS*) levels (*p* > 0.05). However, the levels of toll-like receptor 4 (*TLR4*), a major lipopolysaccharide receptor, were higher in the 0.5% and 1% PWMC groups (*p* = 0.015). Both interferon-γ (*IFN-γ*) and interleukin-1ß (*IL-1ß*) levels decreased significantly (*p* < 0.0001 and = 0.0002, respectively), by about three to five times for *IFN-γ* and 1.1 to 1.6 times for *IL-1ß* (Figure 3A).

The expression of all antioxidant-related mRNA, including heme oxygenase-1 (HO-1), nuclear factor erythroid 2–related factor 2 (Nrf-2), glutamate-cysteine ligase catalytic (GCLC), Gpx, and *SOD-1*, increased significantly following PWMC supplementation (Figure 3B), but while *HO-1, Nrf-2*, *GCLC*, and *Gpx* increased with PWMC dosage, *SOD-1* increased equally in each group (Figure 3B).

## 4. Discussion

Food intake is the only way for animals to gain energy, maintain growth, and survive. Traditionally, due to its low energy content, fiber has not been used in feed [3]. However, research proves that adding fiber to animal feed enhances gut barrier expression, increases antioxidant capacity, and decreases the inflammatory response [16]. There are many polyprotein structures on intestinal epithelial tissue. These structures can block exogenous toxins and bacteria in the intestinal lumen [17]. The immune response is triggered and macrophages are activated when these toxins invade [18]. Due to the complexity of gut microbiota, animals have developed a set of modes that can coexist with microorganisms. Within a single day, animals can adjust the composition of gut microbiota through their diet, further affecting their health and the state of the intestinal barrier [16,17]. Fiber supplementation in animal diets could significantly increase gut barrier function and alter metabolism [16,19]. After PWMC supplementation, the gut barrier in a broiler’s ileum increases, by increments that are positively related to the amount of supplement provided. It was also determined that PWMC supplementation increases the expression of *EAAT3,* one of the major transporters of glutamate and cysteine. Furthermore, under the oxidant stress the *EAAT3* expression would decrease in broilers [20]. In this study, high-fiber PWMC supplementation had no negative impact on broiler growth, and even slightly increased FCR performance in the 0.5% PWMC group during the starter stage; there was no significant difference in the other groups.

In animals, the main tissues for adipose synthesis are the liver and adipocytes. CCAAT-enhancer-binding proteins (*CEBP*) and peroxisome proliferator-activated receptor (*PPAR*) are involved in the early differentiation of adipocytes [21]; however, both genes were downregulated in the PWMC-supplemented groups. *FAS* and *FABP4* are mainly involved in the late differentiation of fat cells and the accumulation of fatty oil droplets [22,23]. The former is an upstream gene for fat synthesis and the latter is one of the major proteins that transport fatty acids [22,23]. Therefore, PWMC could suppress adipocyte differentiation by blocking *CEBP* and *PPAR* but would not affect adipose synthesis. Both KCTD-15 and adiponectin promote adipose metabolism, but adiponectin also promotes leptin production [24,25]. It is well-known that leptin stimulates fat metabolism in animals and enhances muscle-cell development [26]. In this study, both mRNA and protein types adiponectin significantly improved with 1% and 2% PWMC supplementation. While both *ATGL* and *CPT-1* enhance adipose metabolism, the former separates glycerol from fatty acids, while the latter enhances fatty acid metabolism [27,28]. The increase of *ATGL* mRNA expression leads to a decrease in the triglyceride (TG) content in the serum of broilers.

PWMC has a high amount of phenolic compounds. Phenolic compounds, which exist in plant-based ingredients have been regarded as a functional antioxidant component for decades [9,29]. Among them, catechins were regarded as one the most functional compounds on both antioxidant and anti-adipogenesis grounds [8,29]. Past research has pointed out that catechins such as EGCG (210 μg/ g PWMC) can assist in adipose degradation and improve the antioxidant capacity of animals [29]. Traditionally, OXT has been considered a contractile stimulator of smooth muscle that also helps regulate animal mood [30]. However, recent studies have shown that OXT can also help muscle cells take up glucose, enhance fat metabolism, and promote muscle hyperplasia [31]. In addition, OXT can help solve the problem of fat infiltration caused by obesity [31].

PWMC can enhance adipolysis in both the liver and adipocytes, thus compensating for the gene for adipogenesis. As the increase in adipogenesis-related genes is much lower than adipolysis-related gene expression, the overall adipose content in broilers is reduced. Although adipose is rapidly metabolized, broiler weight was still not significantly different from that of the control. This may be due to the transfer of energy from the breakdown of fat to the production of muscle. Previous research also indicates that dietary fiber supplementation could increase broiler muscle content and the slaughter rate of poultry [32]. However, to our best knowledge, there are only a few studies into the cause of increased muscle weight. Although we only measured adipose metabolism-related mRNA and protein expression in this study, we still confirmed that adding extra fiber to the diet can improve the pattern of adipose metabolism, which may be the reason why high fiber diets can promote muscle mass gains in poultry [19].

Animals may suffer from oxidative stress caused by exogenous reactive oxygen species (ROS) which causes incomplete replication of DNA, and induce mutations or cell lesions [33]. After the mature *Pleurotus eryngii* is removed, the remaining culture medium, also known as PWMC, contains many mycelia. Mushrooms have superb antioxidant capacities and can increase antioxidant capacity in animals [5]. Wang et al. [5,34] indicated that mushroom compost can improve the antioxidant capacity of poultry whether in vivo or *in vitro*. Animal antioxidant systems can be classified as enzymatic or non-enzymatic systems. The former contains enzymes that reduce hydrogen peroxide, such as catalase, and the latter contains small molecules that can directly neutralize ROS, such as glutathione (GSH) [35]. Nrf2 is an upstream antioxidant gene that normally binds to Kelch-like ECH-associated protein 1 in the cytoplasm. When *Nrf2* is activated, Nrf2 enters the nucleus and forms a dimer with the Maf protein. It then reacts with the antioxidant responsive element (ARE) and promotes the expression of downstream antioxidant genes, including *GCLC* and *HO-1* [36,37]. Both *GCLC* and *HO-1* are involved in the antioxidant enzymatic system. *GCLC* is a rate-limiting enzyme in the GSH synthesis pathway [38]. GSH can receive electrons from ROS and become reduced glutathione disulfide (GSSG), which can then be reoxidized by Gpx into GSH. As a catalyst, *HO-1* can indirectly reduce the harm caused by H_2_O_2_ by cleaving hemoglobin into biliverdin [39]. Previous research determined that phenols have antioxidant capacities and will activate *Nrf2* expression [40], therefore mushroom additives can improve the enzymatic antioxidant system and enhance animals’ adaptability to oxidative stress [34]. In addition, as a phenolic compound, epigallocatechin (1.493 mg /g PWMC) can enhance the antioxidant capacity of animals by chelating metal ions or increasing antioxidant enzymes [29]. In addition to ROS, Lee et al. [18] pointed out that inflammation is a double-edged sword. Although inflammation can help animals fight pathogens, it may also increase oxidative stress and cause cell damage [18]. Therefore, it is important to appropriately suppress the inflammatory response under non-disease conditions. *IL-1ß*, one of the most upstream inflammatory factors, is involved in upregulating the expression of inflammatory genes like *iNOS* and *NF-κB*, and may lead to the production of reactive nitrogen species [13]. However, suppressed *IL-1ß* performance would not alter the inflammatory response under normal conditions. *IFN-γ* levels are an indicator of infection. When an animal is infected with a virus or bacteria, *IFN-γ* levels increase and activate *NF-κB*, which further increases inflammation. The glucan content in PWMC might decrease *IFN-γ* expression [13]. Overall, PWMC could increase gut barrier function, antioxidant capacity, and alter the adipose metabolism pattern.

## 5. Conclusions

Dietary supplementation with PWMC can significantly change the adipose metabolism pattern in broilers and accelerate adipose degradation. Although higher amounts of PWMC could increase intestinal barrier function and antioxidant capacity, supplementation with 0.5% and 2% PWMC resulted in similar levels of adipose metabolism. Furthermore, the 0.5% PWMC group had better FCR. Therefore, to improve health and growth efficiency in broilers, our results suggest that supplementation with 0.5% PWMC is the most effective way to improve fat metabolism and antioxidant capacity. However, further research is recommended to detect the actual mechanism of fat metabolism and antioxidant capacity of PWMC in broilers.

## Figures and Tables

**Table 1:** Composition and calculated analysis (g/kg as fed) of the basal diet for broilers (1–35 days) ^1^.

Ingredients	Starter Diet	Finisher Diet
	(1–21 Days)	(22–35 Days)
	g/kg	
Corn, yellow	520	572
Soybean meal (CP 44%)	369	247
Full-fat soybean meal	0.00	50.0
Soybean oil	40.6	41.4
Fish meal (CP 65%)	30.0	50.0
Calcium carbonate	16.5	16.5
Monocalcium phosphate	11.2	11.2
_DL_-Methionine	2.00	2.00
_L_-Lysine-HCl	3.70	3.70
NaCl	3.90	3.90
Choline-Cl	0.80	0.80
Vitamin premix ^2^	1.00	1.00
Mineral premix ^3^	1.00	1.00
Total	1000	1000
Calculated nutrient value		
Dry matter, %	88.1	88.2
Crude protein, %DM	23.0	21.0
Crude fiber, %DM ^4^	4.71	3.55
Calcium, %DM	1.11	1.21
Total Phosphorus, %DM	0.68	0.72
Available Phosphorus, %DM	0.46	0.52
Methionine + Cysteine, %DM	0.92	0.89
ME, kcal/kg DM	3050	3175
Chemical analysis value		
Dry matter, %	88.7	89.3
Crude protein, %DM	23.1	21.0
Crude fat, DM%	6.85	7.96
Chemical analysis value	PWMC	
Neutral detergent fiber, %DM	60.1 ± 0.70	
Crude protein, %DM	6.50 ± 0.40	
Ash, %DM	10.2 ± 0.20	

^1^ Con: basal diet for control group; PWMC: *Pennisetum purpureum* Schum No. 2 waste mushroom compost. PWMC was added directly to the basal diet at different percentages. ^2^ Vitamins (premix content per kg diet): Vit. A, 15,000 IU; Vit. D3, 3000 IU; Vit. E, 30 mg; Vit. K3, 4 mg; thiamine, 3 mg; riboflavin, 8 mg; pyridoxine, 5 mg; Vit. B12, 25 μg; Ca-pantothenate, 19 mg; niacin, 50 mg; folic acid, 1.5 mg; and biotin, 60 μg. ^3^ Minerals (premix content per kg diet): Co (CoCO_3_), 0.255 mg; Cu (CuSO_4_·5H_2_O), 10.8 mg; Fe (FeSO_4_·H_2_O), 90 mg; Mn (MnSO_4_·H_2_O), 90 mg; Zn (ZnO), 68.4 mg; Se (Na_2_SeO_3_), 0.18 mg. ^4^ The analysis of crude fiber content in the basal diet, 0.05, 1 and 2% PWMC addition treatments were 4.8, 5.1, 5.3, and 5.7% dry matter (DM) in the starter stage and 3.6, 3.8, 4.1, and 4.5% DM in the finisher stage, respectively.

**Table 2:** The primer sequence of each gene according to Genbank or other research.

Gene Name ^1^	Primer Sequence	Genbank No.
*ß-actin*	F: 5′-CTGGCACCTAGCACAATGAA-3′ R: 5′-ACATCTGCTGGAAGGTGGAC-3′	X00182.1
*TNF-α*	F: 5′-TGTGTATGTGCAGCAACCCGTAGT-3′ R: 5′-GGCATTGCAATTTGGACAGAAGT-3′	NM 204267
*TLR4*	F: 5′-TGCACAGGACAGAACATCTCTGGA-3′ R: 5′-AGCTCCTGCAGGGTATTCAAGTGT-3′	NM_001030693
*NQO-1*	F: 5′-AAGAAGATTGAAGCGGCTGA-3′ R: 5′-GCATGGCTTTCTTCTTCTGG-3′	NM_001277619.1
*NFκB*	F: 5′- CCAGGTTGCCATCGTGTTCC- 3′ R: 5′- GCGTGCGTTTGCGCTTCT -3′	D13719.1
*iNOS*	F: 5′-TACTGCGTGTCCTTTCAACG -3′ R: 5′-CCCATTCTTCTTCCAACCTC-3′	U46504
*IFN-γ*	F: 5′-CTCCCGATGAACGACTTGAG-3′ R: 5′-CTGAGACTGGCTCCTTTTCC-3′	Y07922
*IL-1ß*	F: 5′-GCTCTACATGTCGTGTGTGATGAG-3′ R: 5′-TGTCGATGTCCCGCATGA-3′	NM_204524
*HO-1*	F: 5′-GGTCCCGAATGAATGCCCTTG-3′ R: 5′-ACCGTTCTCCTGGCTCTTGG-3′	NM_205344.1
*Nrf-2*	F: 5′-GGAAGAAGGTGCGTTTCGGAGC-3′ R: 5′-GGGCAAGGCAGATCTCTTCCAA-3′	NM_205117.1
*GCLC*	F: 5′-CAGCACCCAGACTACAAGCA-3′ R: 5′-CTACCCCCAACAGTTCTGGA-3′	XM_419910.3
*GPx*	F: 5′- CAGCAAGAACCAGACACCAA-3′ R: 5′- CCAGGTTGGTTCTTCTCCAG-3′	NM_001163245.1
*SOD-1*	F: 5′- ATTACCGGCTTGTCTGATGG-3′ R: 5′- CCTCCCTTTGCAGTCACATT-3′	NM_205064.155
*Claudin-1*	F: 5′-GGAGGATGACCAGGTGAAGA-3′ R: 5′-TCTGGTGTTAACGGGTGTGA-3′	NM_001013611.2
*MUC-2*	F: 5′-GCTACAGGATCTGCCTTTGC-3′ R: 5′-AATGGGCCCTCTGAGTTTTT-3′	JX284122.1
*Occludin*	F: 5′-GTCTGTGGGTTCCTCATCGT-3′ R: 5′-GTTCTTCACCCACTCCTCCA-3′	NM_205128.1
*ZO-1*	F: 5′-AGGTGAAGTGTTTCGGGTTG-3′ R: 5′-CCTCCTGCTGTCTTTGGAAG-3′	XM_015278975.1
*EAAT3*	F: 5′- ACCCCCTTCTGATCACCTCT-3′ R: 5′- TGAGCATGCTGATTCCAAAG-3′	XM_424930.6
*FFAR2*	F: 5′-GCCCCATAGCAAACTTCT-3′ R: 5′-GGGCAGCCATAAAGAGAG-3′	[15]
*GLUT2*	F: 5′- CCGCAGAAGGTGATAGAAGC-3′ R: 5′- ACACAGTGGGGTCCTCAAAG-3′	NM_207178.1
*SGLT*	F: 5′- CATCTTCCGAGATGCTGTCA-3′ R: 5′- CAGGTATCCGCACATCACAC-3′	NM_001293240.1
*PEPT-1*	F: 5′- CAGGGATCGAGATGGACACT-3′ R: 5′- CACTTGCAAAAGAGCAGCAG-3′	NM_204365.1
*KCTD-15*	F: 5′-TTAAAAACACCCCGTTCTGC-3′ R: 5′-AAAACAAACCAAGCGACCAC-3′	XM_004944237
*Adiponectin*	F: 5′-ACTTTCATGGGCTTCCTCCT-3′ R: 5′-GTCCCACGGAAGTCACTTGT-3′	NM_206991
*ATGL*	F: 5′-CAGCAGGACGTTTGGGTATT-3′ R: 5′-CCACGCAAAGTTGGAGGTAT-3′	EU240627.2
*AMPK-α2*	F: 5′-GGCATTGAGGAAATCAGGAA-3′ R: 5′-CCTGAACCAATGTGTGTTGC-3′	DQ340396
*FAS*	F: 5′-GCTGAGAGCTCCCTAGCAGA-3′ R: 5′-TCCTCTGCTGTCCCAGTCTT-3′	NM_205155
*FABP4*	F: 5′-CAGCATCAATGGTGATGTGA-3′ R: 5′-TCTCTTTGCCATCCCACTTC-3′	NM_204290
*CEBP* *α*	F: 5′-GGAGCAAGCCAACTTCTACG-3′ R: 5′-GTCGATGGAGTGCTCGTTCT-3′	NM_001031459
*CPT-1*	F: 5′-ATCCCAGCTGCAGTGAGTCT-3′ R: 5′-ATTCGCAAGTCAATCCCATC-3′	NM_001012898
*IL-6*	F: 5′-AGGACGAGATGTGCAAGAAGTTC-3′ R: 5′-TTGGGCAGGTTGAGGTTGTT-3′	NM_204628
*PPAR-γ*	F: 5′-GATCGCCCAGGTTTGTTAAA-3′ R: 5′-TGCACGTGTTCCGTTACAAT-3′	NM_001001460
*PPAR-α*	F: 5′-AGGCCAAGTTGAAAGCAGAA-3′ R: 5′-GTCTTCTCTGCCATGCACAA-3′	NM_001001464.1

^1^ TNF-α: Tumor necrosis factor alpha; TLR4: Toll-like receptor 4; NQO-1: NADPH dehydrogenase 1; NFκB: Nuclear factor kappa B p 65; iNOS: Inducible nitric oxide synthases; IFN-γ: Interferon-γ; IL-1ß: Interleukin-1ß; HO-1: Heme oxygenase-1; Nrf-2: Nuclear factor erythroid 2–related factor 2; GCLC: Glutamate-cysteine ligase catalytic; Gpx: glutathione peroxidase; SOD-1: Superoxide dismutase-1; MUC-2: Mucin2; ZO-1: Zonula occludens 1; EAAT3: Excitatory amino acid transporter 3; FFAR2: Free fatty acid receptor 2; GLUT2: glucose transporter 2; SGLT: sodium-dependent glucose cotransporters 1; PEPT-1: Peptide transporter 1; KCTD-15: Potassium channel tetramerization domain-containing 15; ATGL: Adipose triglyceride lipase; AMPK-α2: 5′-AMP-activated protein kinase catalytic subunit alpha-2; FAS: Fatty acid synthase; FABP4: Fatty acid binding protein 4; CEBPα: CCAAT-enhancer-binding proteins-alpha; CPT-1: Carnitine palmitoyltransferase I; IL-6: Interleukin-6; PPAR-γ: Peroxisome proliferator-activated receptor gamma; PPAR-α: Peroxisome proliferator-activated receptor alpha.

**Table 3:** The functional chemical composition analysis in PWMC.

Items	PWMC
Functional component analysis	
Crude triterpenes (mg/ g DM ^1^)	6.25 ± 0.37
Total phenol contents (GAE ^2^ mg/ g DM)	1.84 ± 0.05
Total flavonoids (QE ^3^ mg/ g DM)	1.20 ± 0.26
Phenol-like chemical analysis (μg/ g DM)	
Gallic acid	114.0 ± 2.7
Gallocatechin	1035.0 ± 8.0
Epigallocatechin	1493.0 ± 14.0
Catechin	7.9 ± 0.6
Caffeic acid	113.0 ± 0.5
Epicatechin	229.8 ± 2.0
Epigallocatechin gallate	210.2 ± 0.1
Epicatechin gallate	78.1 ± 2.8
Catechin gallate	401.2 ± 5.1

^1^ DM: Dry matter; ^2^ GAE: Gallic acid equivalent; ^3^ QE: Quercetin. (n = 5).

**Table 4:** The growth performance of 35-day-old broilers following dietary PWMC ^1^ supplementation.

Items	Treatments				SEM ^2^	*p* Value
	Con	0.5% PWMC	1% PWMC	2% PWMC		
1–21 d						
Body weight, g/bird	925	956	939	919	30.2	0.848
Weight gain, g/bird	880	907	890	871	30.1	0.853
Feed consumption, g/bird	1197	1161	1184	1219	38.1	0.691
FCR	1.36 ^a^	1.28 ^b^	1.33 ^ab^	1.40^a^	0.03	0.037
22–35d						
Body weight, g/bird	2019	2103	2021	2009	30.7	0.184
Weight gain, g/bird	1091	1148	1081	1089	56.7	0.335
Feed consumption, g/bird	2007	2204	2142	2114	107	0.641
FCR	1.84	1.92	1.98	1.94	0.09	0.742
1–35 d						
Feed consumption, g/bird	3213	3123	3135	3176	30.9	0.190
Weight gain, g/bird	1971	2055	1972	1960	122	0.965
FCR	1.63	1.52	1.59	1.62	0.058	0.588

^1^ Con: basal diet for control group; PWMC: *Pennisetum purpureum* Schum No. 2 waste mushroom compost. PWMC was added directly to the basal diet at different percentages. ^2^ SEM: Standard error of mean. ^a,b^ Means within the same rows without the same superscript letter are significantly different (*p* < 0.05).

**Table 5:** Jejunum and ileum morphology of 35-day-old broilers following PWMC ^1^ supplementation.

Items	Treatments				SEM ^2^	*p* Value
	Con	0.5% PWMC	1% PWMC	2% PWMC		
Jejunum						
Villus height (μm)	1212	1301	1367	1467	69.9	0.109
Crypt depth (μm)	192	208	203	190	9.37	0.496
*Tunica muscularis* (μm)	152 ^b^	224 ^a^	204 ^ab^	162^ab^	22.1	0.010
Villus:crypt	6.32 ^b^	6.29 ^b^	6.73 ^b^	7.75^a^	0.24	0.002
Ileum						
Villus height (μm)	1166	1110	1102	1038	38.3	0.175
Crypt depth (μm)	171	159	168	158	10.8	0.798
*Tunica muscularis* (μm)	166	137	144	173	25.6	0.712
Villus:crypt	6.84	6.99	6.58	6.99	0.52	0.932

^1^ Con: basal diet for control group; PWMC: *Pennisetum purpureum* Schum No. 2 waste mushroom compost. PWMC was added directly to the basal diet at different percentages. ^2^ SEM: Standard error of means. ^a,b^ Means within the same rows without the same superscript letter are significantly different (*p* < 0.05).

**Table 6:** Serum characteristics of 35-day-old broilers following PWMC ^1^ supplementation.

Items ^3^	Treatments				SEM ^2^	*p* Value
	Con	0.5% PWMC	1% PWMC	2% PWMC		
GLU (mg/dL)	276 ^a^	243 ^b^	260 ^ab^	224 ^c^	6.10	<0.001
MDA (uM)	10.1	15.2	13.3	11.4	1.70	0.234
Protein						
SGOT (U/ L)	332	286	273	342	41.0	0.568
SGPT (U/ L)	4.20	4.00	4.50	6.00	0.690	0.184
Alk-P (IU/ L)	1086	937	1366	1282	277	0.694
Gpx (nmol/min/mL)	115	126	129	115	9.59	0.641
SOD (mU/ mL)	524 ^b^	865 ^a^	843 ^a^	879 ^a^	20.7	<0.001
TNF-α (pg/mL)	285 ^a^	162 ^c^	176 ^c^	232 ^b^	16.6	0.001
Oxytocin (pg/mL)	259 ^b^	447 ^b^	874 ^a^	963 ^a^	93.9	0.002
Adiponectin (ng/mL)	5948 ^c^	5709 ^c^	11820 ^a^	7938 ^b^	303	<0.001
Corticosterone (pg/mL)	133	121	126	118.2	3.45	0.064
Lipid						
CHOL (mg/dL)	125	124	137	122	7.00	0.379
TG (mg/dL)	54.8 ^a^	38.3 ^b^	43.7 ^ab^	40.2 ^b^	2.30	<0.001
HDL-C (mg/dL)	78.0	76.2	83.7	73.2	3.70	0.263
LDL-C (mg/dL)	42.2	42.5	48.0	42.5	3.40	0.577

^1^ Con: basal diet for control group; PWMC: *Pennisetum purpureum* Schum No. 2 waste mushroom compost. PWMC was added directly to the basal diet at different percentages. ^2^ SEM: Standard error of means. ^3^ GLU: Glucose; MDA: malondialdehyde; SGOT: Serum glutamic-oxaloacetic transaminase; SGPT: Serum glutamic-pyruvic transaminase; Alk-P: Alkaline phosphatase; Gpx: Glutathione peroxidase; SOD: Superoxide dismutase; TNF-α: Tumor necrosis factor alpha; CHOL: Cholestenone; TG: Triglyceride; HDL-C: High density lipoprotein-cholestenone; LDL-C: Low density lipoprotein-cholestenone. ^a,b,c^ Means within the same rows without the same superscript letter are significantly different (*p* < 0.05).

## Abbreviations

PWMC	*Pennisetum purpureum* schum No.2 waste mushroom compost
PP	*Pennisetum purpureum* schum No.2
OXT	Oxytocin
TNF-α	Tumor necrosis factor alpha
Gpx	Glutathione peroxidase
SOD	Superoxidase dismutase
MDA	Malondialdehyde
TLR4	Toll-like receptor 4
NQO-1	NADPH dehydrogenase 1
NFκB	Nuclear factor kappa B p 65
iNOS	Inducible nitric oxide synthases
IFN-γ	Interferon-γ
IL-1ß	Interleukin-1ß
HO-1	Heme oxygenase-1
Nrf-2	Nuclear factor erythroid 2–related factor 2
GCLC	Glutamate-cysteine ligase catalytic
MUC2	Mucin2
ZO-1	Zonula occludens 1
EAAT3	Excitatory amino acid transporter 3
FFAR2	Free fatty acid receptor 2
GLUT2	Glucose transporter 2
SGLT	Sodium-dependent glucose cotransporters 1
PEPT-1	Peptide transporter 1
KCTD-15	Potassium channel tetramerization domain-containing 15
ATGL	Adipose triglyceride lipase
AMPK-α2	5′-AMP-activated protein kinase catalytic subunit alpha-2
FAS	Fatty acid synthase
FABP4	Fatty acid binding protein 4
CEBPα	CCAAT-enhancer-binding proteins-alpha
CPT-1	Carnitine palmitoyltransferase I
IL-6	Interleukin-6
PPAR-γ	Peroxisome proliferator-activated receptor gamma
PPAR-α	Peroxisome proliferator-activated receptor alpha
FCR	Feed conversion rate
SEM	Standard error of mean
GLU	Glucose
SGOT	Serum glutamic-oxaloacetic transaminase
SGPT	Serum glutamic-pyruvic transaminase
Alk-P	Alkaline phosphatase
CHOL	Cholestenone
TG	Triglyceride
HDL-C	High density lipoprotein-cholestenone
LDL-C	Low density lipoprotein-cholestenone
ROS	Reactive oxygen species
GSH	Glutathione
ARE	Anti-oxidant responsive element
GSSG	Glutathione disulfide
